# Exploring the Stress and Strengths Arising From the Complexities Existing Within Grandfamilies

**DOI:** 10.1093/geroni/igaa057.237

**Published:** 2020-12-16

**Authors:** Catherine Tompkins, Alice Zic, Ellie Carlson, Ali Purvis, Loriena Yancura, Danielle Nadorff

**Affiliations:** 1 George Mason University, Fairfax, Virginia, United States; 2 University of Hawai'i, Honolulu, Hawaii, United States; 3 Mississippi State University, Mississippi State, Mississippi, United States

## Abstract

The opioid crisis and other social problems continue to increase the number of grandparent-headed households in the U.S. There are challenges and benefits that result from grandparents parenting their grandchildren. Grandparents often report the joy in watching their grandchildren grow but also report on the complexities that may lead to stress. Two-hundred forty-one grandparents were recruited using Qualtrics’ Online Panel Service. In addition to a standardized perceived stress scale and demographic questions, participants responded to open-ended questions related to the benefits and challenges of residing within a grandparent-headed household. This presentation focuses on comparing demographics, perceived stress, benefits and challenges of 108 current grandparent caregivers to 133 grandparents who were no longer the head of household at the time of the survey. Grandparents who currently were raising their grandchildren had a higher perceived stress score (p=.03) compared to grandparents who had raised their grandchildren in the past. An exploration of the demographic variances and responses to the open-ended questions, will begin to explain this statistically significant difference in reported stress. An additional complexity arising for a subsample of 10 current grandparents raising grandchildren, included simultaneously caregiving for an older adult relative. It is imperative to study the complexities existing within grandfamilies, from the perspectives and experiences of the grandparent caregiver, and develop interventions to reduce stress and increase the grandparents’ ability to cope with situational, emotional and relationship changes.

